# Imaging G-Quadruplex Nucleic Acids in Live Cells Using Thioflavin T and Fluorescence Lifetime Imaging Microscopy

**DOI:** 10.1021/acs.analchem.4c04207

**Published:** 2024-12-11

**Authors:** Tigerlily Bradford, Peter A. Summers, Aatikah Majid, Petr S. Sherin, Jeff Yui Long Lam, Savyasanchi Aggarwal, Jean-Baptiste Vannier, Ramon Vilar, Marina K. Kuimova

**Affiliations:** † Molecular Sciences Research Hub, Department of Chemistry, Imperial College London, London W12 0BZ, U.K.; ‡ Telomere Replication and Stability Group, MRC London Institute of Medical Sciences, London W12 0NN, U.K.; § Institute of Clinical Sciences, Faculty of Medicine, Imperial College London, London W12 0NN, U.K.

## Abstract

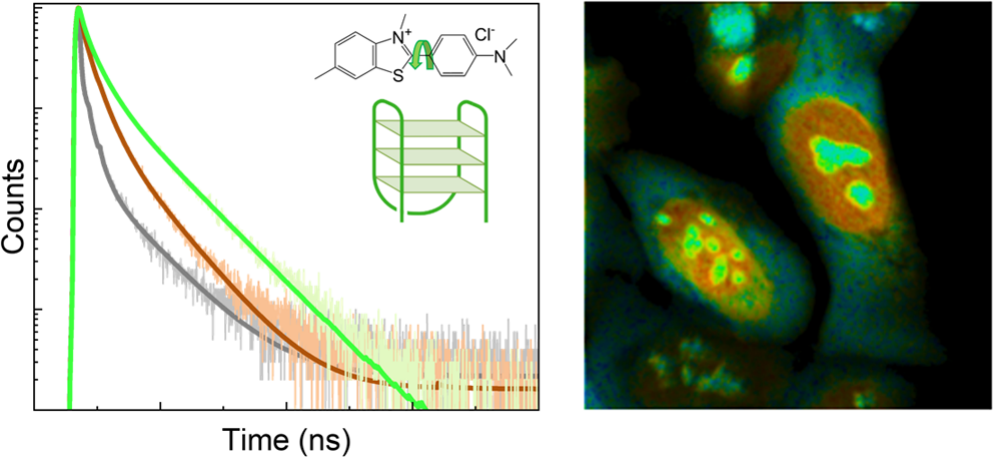

Visualization of guanine-rich oligonucleotides that fold into G-quadruplex (G4) helical structures is of great interest in cell biology. There is a large body of evidence that suggests that these noncanonical structures form *in vivo* and play important biological roles. A promising recent development highlighted fluorescence lifetime imaging microscopy (FLIM) as a robust technique for the direct and quantitative imaging of G4s in live cells. However, this method requires specialized, bespoke synthetic dyes that are not widely available. Herein, we demonstrate that the fluorescence lifetime of commercially available environmentally sensitive dyes Thioflavin T (**ThT**) and Thiazole Orange (**TO**) is strongly dependent on the type of DNA topology they bind to, with G4s showing long and distinctive decay times that should allow G4 detection in the biological environment. We applied this observation to visualize G4s in live U2OS cells using FLIM of **ThT**, upon alteration in G4 levels due to competitive binding or nuclease treatment of cells.

## Introduction

G-Quadruplexes (G4s) are noncanonical four-stranded nucleic acid secondary structures that form in guanine-rich regions of the genome. G4s are believed to play important roles in biological processes, such as transcription, replication, and telomere maintenance, as well as in disease development,^1−3^ and in proliferation of viral infections.^4,5^ The biological significance of G4s has stimulated the development of a broad range of optical probes, aiming to detect their formation.^6−9^ One of the key approaches for G4 visualization is *via* antibodies, which can be used in immunostaining of fixed cells followed by fluorescence imaging.^10−12^ However, this technique is not suitable for studying dynamic processes in live cells.

A plethora of small-molecule optical probes have been developed for the detection of G4s,^6,7,9^ but practically all of them rely on a luminescence intensity switch-on upon interactions with G4s. While this phenomenon is very useful to study G4 formation *in vitro*, it is generally unsuitable for cellular studies. Indeed, fluorescence intensity is dependent on the local concentration of the probe and most switch-on probes will also respond with increased intensity to large levels of duplex DNA, present in the nuclear environment alongside a low concentration of G4s.^7,9^ One elegant example where the problem of concentration-dependent intensity measurements has been overcome is *via* the use of single-molecule measurements, where the probe SiR-PyPDS lights up upon individual molecules binding to G4s in cell nuclei.^8^ However, this method is applicable only for the monitoring of single-molecule dynamics.

An alternative solution is the use of probes that display variations in their time-resolved fluorescence decays (characterized by a fluorescence lifetime) upon binding to different DNA topologies. Our group^13−16^ and others^17−19^ have demonstrated that this type of probes can be used in combination with fluorescence lifetime imaging microscopy (FLIM) to visualize G4s in live cells, even in the presence of a large excess of duplex DNA, due to the concentration-independent nature of fluorescence lifetime measurements. However, only a limited number of such probes have been reported and while some of them work in live cells,^11,12,15,16^ others only allow fixed-cell staining.^13,14,17^ None of the fluorescence-lifetime-based probes are available commercially but instead require multistep synthesis and extensive purification that is typically not available for chemical biology laboratories with interest in G4s.

Herein, we explore the possibility of using two commercially available conformationally flexible dyes for FLIM-based detection of G4 in live cells, Thiazole Orange (**TO**) and Thioflavin T (**ThT**).

## Experimental Methods

### General Procedures

All commercial chemicals and solvents were used as received unless stated otherwise. Stock solutions of 1-methyl-4-[(3-methyl-2(3*H*)-benzothiazolylidene)methyl]quinolinium *p*-tosylate (thiazole orange, **TO**, Glentham Life Sciences, 98% purity) and 2-[4-(dimethylamino)phenyl]-3,6-dimethyl-1,3-benzothiazol-3-ium chloride (Thioflavin T, **ThT**, Sigma-Aldrich, Calbiochem) were prepared in DMSO at a concentration of 5 mM. Ni-Salphen was synthesized according to the published literature procedure.^20^

DNA oligonucleotides (see Table S1, Supporting Information (SI), for sequences used) were purchased from Eurogentec (RP Cartridge purification) and used as received. The oligonucleotides were dissolved in 10 mM lithium cacodylate buffer at pH 7.3. KCl was added to a final concentration of 100 mM, and the resulting oligonucleotide solution was annealed at 95 °C for 10 min. Calf thymus DNA (CtDNA, Sigma) was dissolved in the same cacodylate buffer, and KCl was added to a final concentration of 100 mM. All oligonucleotide concentrations were determined in salt-free buffer (before any annealing) using the molar extinction coefficients listed in Table S1 (SI) and 13,200 M^–1^ cm^–1^ (base pair for CtDNA). Concentrations of G4s are per strand while concentrations of dsDNA are per base pair. Single strands of ds17 DNA were combined at a molar ratio of 1:1 to make double-stranded ds17.

Fluorescence spectra were recorded by using a Fluoromax4 spectrofluorimeter (Jobin Yvon; Horiba). Absorbance spectra were recorded using an 8453 UV Visible Spectroscopy System (Agilent). Quartz cuvettes with a 10 mm optical path were used for all spectroscopic measurements.

### Time-Correlated Single Photon Counting (TCSPC)

Time-resolved fluorescence decays were obtained using a DeltaFlex time-correlated single photon counting (TCSPC) device (Jobin Yvon, Horiba) equipped with a 404 nm NanoLED as an excitation source for **ThT** and 467 nm source for **TO** (pulse width <200 ps, HORIBA) with a 100 ns time window and 4096-time bins.

Decays were detected at λ_em_ = 490 nm (±8 nm) after passing through a 420 nm long-pass filter to remove any scattered excitation pulse for **ThT** and λ_em_ = 530 nm (±8 nm) after passing through a 495 nm long-pass filter to remove any scattered excitation pulse for **TO**. Decays were accumulated to 10,000 counts at the peak of fluorescence decay. A neutral density filter was used for the instrument response function (IRF) measurements by using a *Ludox* solution, detecting the emission at the excitation wavelength. Decay traces were fitted by iterative reconvolution to the equation *I*(*t*) = *I*_0_(α_1_ e^–*t*/τ_1_^ + α_2_ e^–*t*/τ_2_^ + α_3_ e^–*t*/τ_3_^), where α_1_, α_2_, and α_3_ are variables normalized to unity. The intensity-weighted average lifetime (τ_w_) was calculated using the following equation:
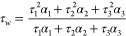
1

A prompt shift was included in the fitting to consider differences in the emission wavelength between the IRF and the measured decay. The goodness of fit was judged by consideration of the deviations from the model *via* a plot of weighted residuals. We found that decay traces for **TO** (c-myc-bound) are best fitted to a biexponential decay equation (*a*_3_ = 0).

### Oligonucleotide Titrations

An aliquot of the probe from the 5 mM DMSO stock solution was diluted to 5 μM for in cellular measurements or 2 μM for *in vitro* measurements using 10 mM lithium cacodylate buffer (pH 7.3) supplemented with 100 mM KCl. The UV/visible/fluorescence spectra and/or TCSPC time-resolved decays were recorded (where applicable). Increasing amounts of the oligonucleotide under study were added, maintaining a constant concentration of the probe. After each addition, the mixture was left to equilibrate for 30 s (a time determined to be sufficient for equilibrium to occur) before the corresponding photophysical measurements were recorded. Fluorescence spectra were integrated between 500 and 650 nm and the integrated intensity was normalized against the absorption at the excitation wavelength.

The fluorescence “switch-on” values for **ThT** and **TO** were calculated as *F*/*F*_0_, where *F* is calculated from the asymptotic value of the binding fit, and *F*_0_ is the initial point on the binding curve, *i.e.*, the fluorescence intensity of the buffer before the addition of oligonucleotide. N.B, these values include correction for absorption at the excitation wavelength. We cannot exclude the presence of a small undetectable amount of aggregated dyes that may affect the switch-on values, as both **ThT** and **TO** are known to aggregate in aqueous solutions.^21,22^

### General Cell Culture

Human Bone Osteosarcoma Epithelial Cells (U2OS, from ATCC) were grown in high-glucose Dulbecco’s modified Eagle’s medium (DMEM) containing 10% fetal bovine serum (FBS) at 37 °C with 5% CO_2_ in humidified air.

For all microscopy experiments, U2OS cells were seeded at 4 × 10^4^ (250 μL, 0.8 cm^2^) in 8-well chamber slides (Lab-Tek Nunc II, part number 155411, Thermo Fisher Scientific, Germany) and allowed to grow for 24 h in high-glucose DMEM.

For live cell experiments, the media was removed, and cells were washed twice with PBS before being incubated with dye solution (2–5 μM in DMEM) for 1–24 h.

For Ni-Salphen displacement experiments, the DMEM containing the dye was removed. Live cells were washed with PBS before being incubated with Ni-Salpen solution (1 μM in DMEM) for 1 h before being washed out and imaged using the conditions below.

For DNase/RNase experiments, the media was removed and cells were washed with cold PBS (×3) before being incubated with 4% formaldehyde in PBS solution for 8 min for fixation. The fixed cells were then further washed with cold PBS (×3). Cells were then treated with either DNase (60 units per well, TurboDNase, Simga-Alrich) or RNase A (0.1 μg/mL, Invitrogen) for 1 h at 37 °C. After this, **ThT** (5 μM in PBS) was added, and the cells were imaged using the conditions below.

### Confocal Imaging

1024 × 1024 resolution were collected using an inverted confocal laser scanning microscope (Leica TCS SP5 II, Leica Microsystem Ltd., Germany). The dye emission was collected at (470–620 nm) following excitation from an internal Ar^+^ laser at 458 nm.

### Fluorescence Lifetime Imaging Microscopy (FLIM)

FLIM images of 256 × 256 pixels were obtained using time-correlated single photon counting (TCSPC) on an inverted confocal laser scanning microscope (Leica TCS SP5 II) with a TCSPC SPC830 single photon counting card (Becker & Hickl GmbH). Internal FLIM detector PMH-100 (Becker & Hickl, Germany), synchronized to a Ti:sapphire pulsed laser source (680–1080 nm, 80 MHz, 140 fs, Chameleon Vision II, Coherent Inc., Germany) or a diode pulsed laser (477 nm, 20/50/80 MHz, 250 ps, BDL-488-SMN, Becker & Hickl, Germany) were used.

Excitation was performed either at 477 nm (one-photon excitation conditions) or at 850 nm (two-photon excitation), and the emission was measured between 500 and 620 nm through an airy 1 pinhole (one-photon excitation at 477 nm) or 440–620 nm with an open pinhole (two-photon excitation at 850 nm). The acquisition time was varied in the range of 100–200 s depending on the emission intensity from each condition. For all live cell imaging, cells in 8-well chamber slides were heated by a thermostat (Lauda GmbH, E200) to 37 (±0.5)°C, and kept under an atmosphere of 5% CO_2_. A 100× (N.A. 1.4) HCX PL APO CS oil immersion objective lens with a correction collar (11506279, Leica Microsystem Ltd., Germany) was used to collect images at 256 × 256 resolution. The IRF used for deconvolution was recorded by using the reflection of the excitation beam from crystals of urea growth on the glass cover slide.

FLIM data sets were fitted using the FLIMFit software (Warren et al.) or SPCImage v.8.3 software (Becker & Hickl, Germany) to a triexponential function, and the intensity-weighted lifetime (τ_w_) was calculated using eq 1. 3 × 3 square binning (bin 1) was used to increase signal strength for images recorded at 256 × 256 resolution. A scatter parameter was added to the decay fitting to account for the scattered excitation light. Before fitting, a mask was applied to the images to analyze individual cell nuclei staining, where indicated. A threshold was applied to the average of each nucleus to require a minimum of 500 counts at the peak of the decay and a goodness of fit measured by χ^2^ of less than 2.

## Results and Discussion

### *In Vitro* Assessment of TO and ThT as G4 Probes

Thiazole Orange (**TO**) and Thioflavin T (**ThT**) are known to strongly change their fluorescence properties upon binding to DNA. **TO** has been widely used as a nucleic acid switch-on probe due to its very large increase in fluorescence intensity on binding to DNA in solution.^21,23^ This phenomenon was previously attributed to the ability of **TO** to rotate around the central bond, which enables efficient nonradiative decay that leads to quenching, while binding to DNA disrupts the rotation and enhances emission.^23^**ThT** is also known to have a large degree of conformational flexibility that affects its fluorescence, which is widely used as an indicator for the formation of fibrillar amyloid fibers.^24^ Additionally, **ThT** can be used as a DNA/RNA binder and the switch-on ability of this optical probe is well documented for G4 DNA.^25−28^

For both these probes, the variation in DNA binding mode (end-stacking onto G4s *vs* intercalation with duplex DNA) determines the fluorescent properties of the bound dye.^9,26^ Since literature data^21,27^ suggests the conformations of **TO** and **ThT** are different upon binding to G4s as compared to duplex DNA, we hypothesized that they would display distinct fluorescence lifetimes when binding to different DNA topologies. This hypothesis is supported by published data for **ThT**, which showed a strong fluorescence lifetime dependence upon binding to various DNA sequences,^27^ although a specific trend for G4 *vs* duplex DNA was not identified.

**Figure 1:**
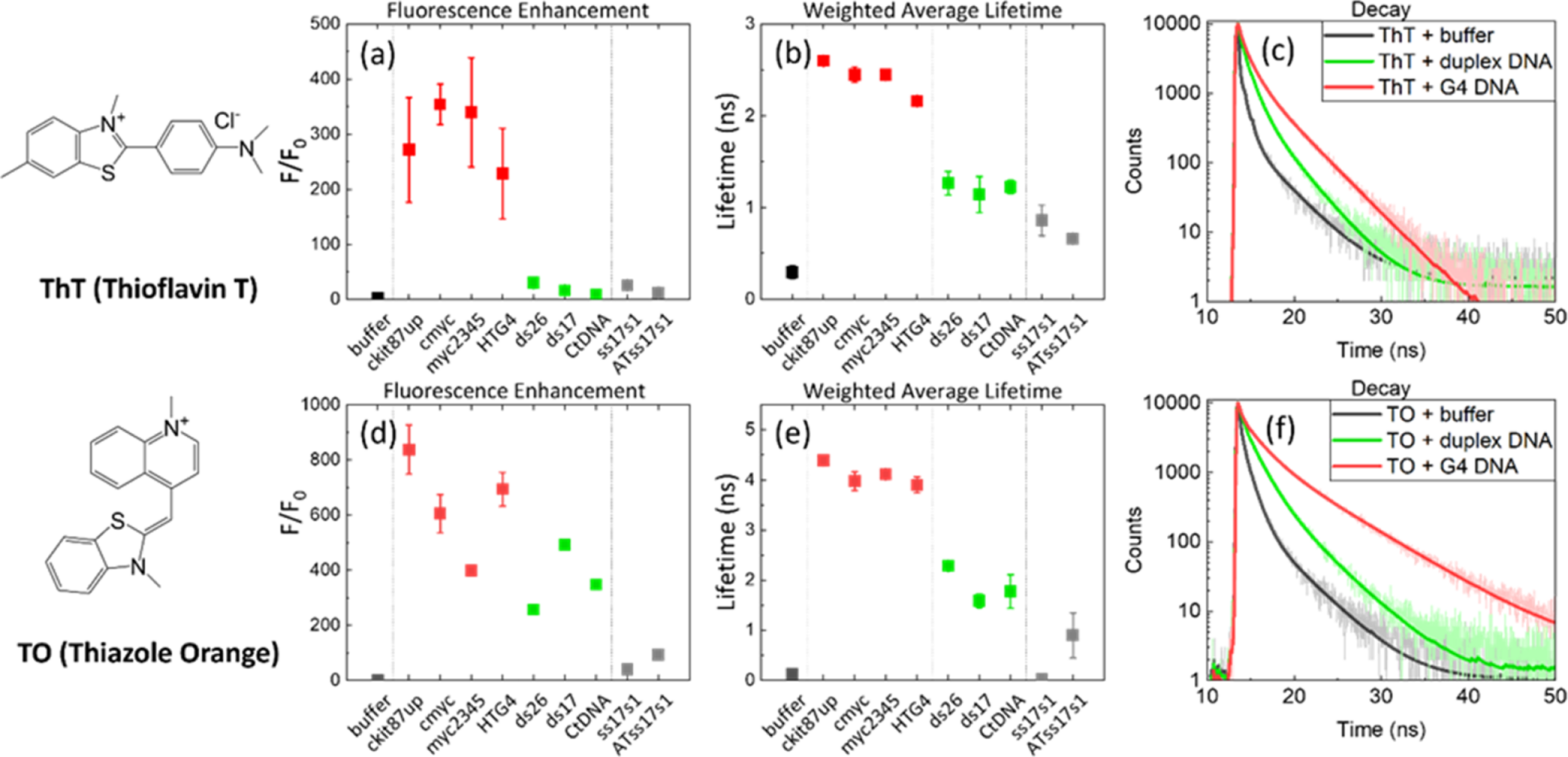
Fluorescence data for Thioflavin T (**ThT**, top) and Thiazole Orange (**TO**, bottom) upon interaction with DNA. Chemical structures of the dyes (left; **TO**’s counterion, i.e., *p*-tosylate, not shown for clarity). Fluorescence intensity enhancement of 2 μM **ThT** (a) and **TO** (d) upon interaction with excess of various DNA topologies (10 μM of G4 and 40 μM of ds/ssDNA); and corresponding weighted average lifetimes (b and e). For **TO** λ_ex_ = 467 nm, and for **ThT** λ_ex_ = 404 nm, for emission enhancement λ_em_ = 500–800 nm, for time-resolved traces λ_em_ = 530 ± 8 nm for **TO** and λ_em_ = 490 ± 8 nm for **ThT**; (a, b, d, e) show the averaged results of at least three repeats per condition. Representative time-resolved decay traces together with triexponential fits are shown in (c) **ThT** and (f) **TO** for buffered solutions (gray), duplex (green), and G4 DNA (red).

Thus, we measured fluorescence spectra and time-resolved decays of **TO** and **ThT** when bound to an excess of various topologies of DNA in solution (Figures 1 and S1–S2). As was expected, a significant increase in fluorescence intensity^21,25−28^ and lifetime was observed for both dyes upon binding to DNA. Complex triexponential fluorescence decays were observed for both dyes with all topologies, Tables S2 and S3, with fitting parameters that are broadly consistent with a small selection of data reported previously.^27,29^ Presumably, this increase in the lifetime is due to a decrease in intramolecular rotation upon binding.

Broadly consistent with the literature data,^21,23^**TO** showed a 300- to 400-fold enhancement upon binding to duplex DNA and 600- to 850-fold enhancement upon binding to G4 DNA (with the exception of myc2345). While G4 binding results in a significantly enhanced fluorescence compared to duplex binding, this increase alone is not sufficient for unambiguous detection of G4 *in cellulo*, due to a large excess of duplex DNA present and identical spectral features generated by both binding events.

Importantly, we detected large and highly statistically significant differences in **TO**’s average fluorescence lifetimes, τ_w_, upon binding to different topologies: 1.58–2.28 ns for duplex DNA and 3.90–4.60 ns for G4 GNA (Figures 1e and S3). This significant difference (*p* < 0.001) with no overlap in values of fluorescence lifetimes when bound to different DNA topologies makes **TO** a promising candidate for the detection of G4 in cells. While the fluorescence intensity of a probe increases as a function of increasing probe concentration, the fluorescence lifetime is an intrinsically ratiometric parameter, which is concentration-independent. Thus, a large distinction in the probe’s lifetimes upon binding to G4 and duplex DNA can serve as a concentration- and uptake-independent measure of the presence of G4s, which is particularly suitable for cellular measurements *via* FLIM.

We also note that a significantly smaller experimental error was seen in the lifetime data as compared to the intensity data for both probes, Figures 1 and S3. One potential reason could be an excitation wavelength-dependent aggregation of **TO** in aqueous solutions,^22^ which is hard to detect due to aggregates being nonemissive. This data further highlights lifetime as a superior parameter for G4 detection in cells, as compared to the fluorescence intensity.

Next, we tested the fluorescence enhancement upon binding of **ThT** to various DNA topologies. Consistent with previous literature data,^25^**ThT** showed up to 50-fold fluorescence enhancement upon binding to duplex DNA, and 200- to 400-fold enhancement upon binding to G4 DNA, with a statistically significant difference of *p* < 0.001, Figure S3. While overall, the fluorescence intensity enhancement of **ThT** upon G4 binding was lower compared to **TO**, the most noteworthy feature was the contrast between G4s and duplex DNA. This can be seen as a major advantage (which was utilized in previous intensity-based fluorescence studies),^26^ allowing a more sensitive method of G4 detection in the background of excess duplex DNA. However, similarly to **TO**, the **ThT** fluorescence enhancement alone is not sufficient for quantitative imaging of G4 in cells, due to an unknown cellular uptake and a large excess of duplex DNA in the cellular environment, compared to G4s.

We detected significant changes (*p* < 0.001) in average fluorescence lifetimes (τ_w_) of **ThT** upon binding to various DNA topologies (Figure 1b,c and Table S3 and Figure S3), which was 1.14–1.26 ns in the presence of the excess of duplex DNA and 2.16–2.60 ns with G4 DNA. Taken together, its selective fluorescence enhancement and distinctive lifetime observed upon binding to G4 *vs* duplex DNA indicate that **ThT** is a promising candidate for a FLIM-based probe to visualize G4 DNA in cells. While **ThT** is known to show higher lifetimes upon increased viscosity^30^ (which could be a contributing factor in the cellular environment), we demonstrated that in the presence of G4 DNA, viscosities between 1 and 50 cP, which can be expected in the cellular nucleus,^31^ have no effect on the observed time-resolved fluorescence decays of **ThT** (Figure S7).

### Efficiency of G4 Discrimination *vs* Duplex DNA

**Figure 2:**
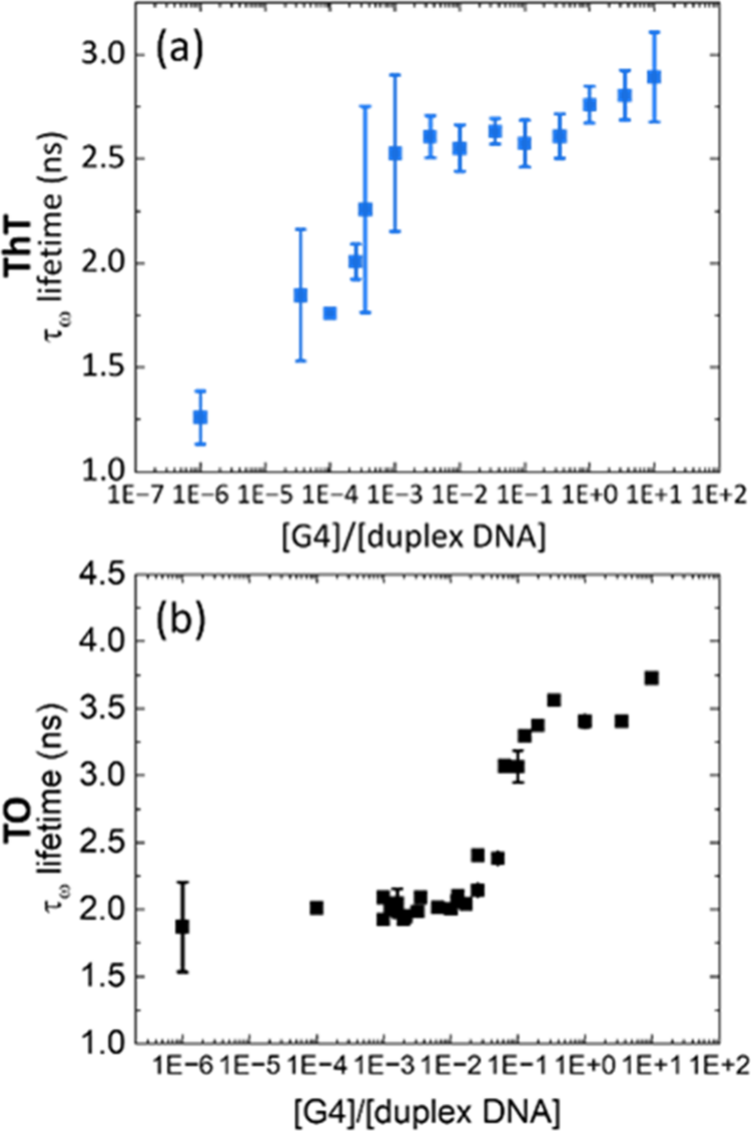
Effect of the G4:duplex DNA ratio on the intensity-weighted averaged lifetimes (τ_w_) of **ThT** (a) and **TO** (b). See Figure S9 for representative time-resolved decays. The data was recorded with 2 μM **ThT**, λ_ex_ = 404 nm, λ_em_ = 490 ± 32 nm and 2 μM **TO**, λ_ex_ = 467 nm, λ_em_ = 530 ± 32 nm in the presence of c-myc (representative G4) and CtDNA (representative duplex). The data are the average of three independent repeats. The points labeled *x* = 1 × 10^–6^ are recorded for pure duplex DNA.

Given that the ratio of duplex DNA to G4 DNA in cell nuclei is largely shifted toward duplex DNA, we next evaluated the lifetime response of **TO** and **ThT** in the presence of both types of DNA with varying ratios, from 1 × 10^–4^:1 to 10:1 G4 *vs* duplex DNA (Figures 2 and S8). We first measured the time-resolved decays of the dyes in the presence of pure duplex CtDNA (the points labeled *x* = 1 × 10^–6^ in Figure 2) and progressively added increasing concentrations of *c-myc* G4 DNA, while maintaining the constant concentration of the dyes. It was observed that upon addition of increasing amounts of G4 DNA to a solution of duplex DNA, the average lifetime increased in a sigmoidal manner for both probes, with inflection points observed at approximately 0.1:1 (**TO**) and 1 × 10^–3^:1 (**ThT**).

This data clearly shows that **ThT** is better suited for G4 detection in the presence of a large excess of duplex DNA, as it is able to detect G4s by its lifetime at less than 1:1000 G4/duplex. In contrast, **TO** could only detect G4s (*via* fluorescence lifetime) at a *ca.* 1:10 G4/duplex DNA ratio (see Figure 2). This difference between the two dyes reflects the high selectivity in the binding of **ThT** to G4 *vs* duplex.^27^ A larger fluorescence enhancement upon binding to G4 compared to duplex (Figure 1) is a favorable factor too, allowing one to visualize G4s more clearly even when a large background of duplex DNA is present.

### *In Cellulo* Assessment of TO and ThT as G4 Probes

**Figure 3:**
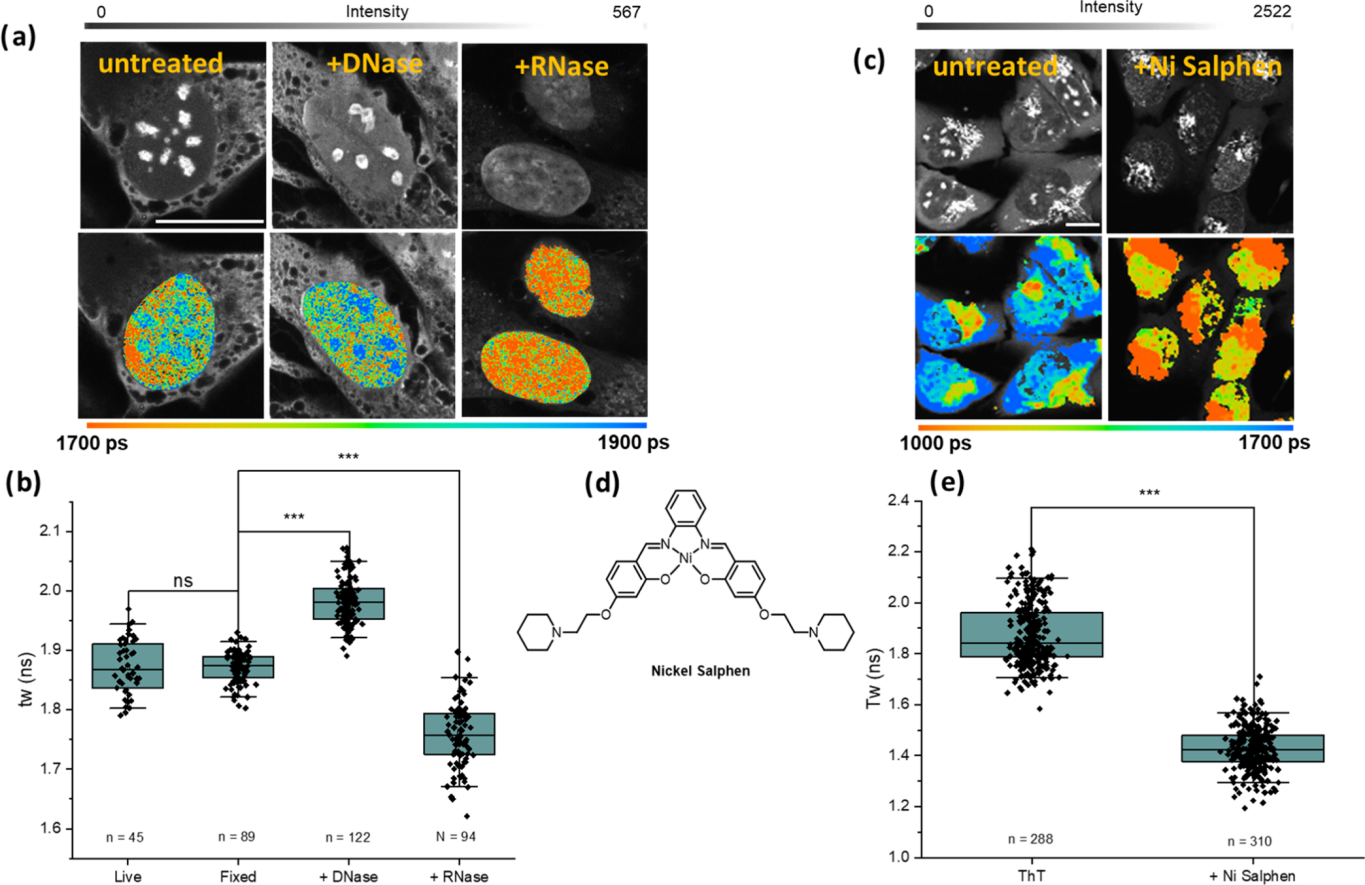
FLIM data from U2OS cells incubated for 1 h with 5 μM **ThT**. (a) Intensity (top) and FLIM (bottom) images of fixed cells that were untreated or incubated with DNase and RNaseA, scale bar 20 μm, gray scale on top gives fluorescence counts per pixel, rainbow scale on the bottom gives weighted average lifetimes per pixel; (b) weighted average lifetime (τ_w_) of nuclei recorded in live cells, compared to fixed and treated cells shown in (a); (c) intensity (top) and FLIM (bottom) images of live cells that were untreated or incubated with a strong G4 binder Ni-Salphen for 1 h, scale bar 20 μm; (d) the chemical structure of Ni-Salphen; (e) weighted average lifetime (τ_w_) of nuclei of untreated live cells as compared to those incubated with Ni-Salphen. Results from n cells as stated below each box, two independent repeats in (b) and three independent repeats in (e). Whiskers in the box plot represent 5–95% of the data while the box shows 25–75% of the data. Results are statistically significant as calculated by two-sample *t* test, *** = *p* < 0.001. is shown in (e); λ_ex_ = 850 nm, λ_em_ = 440–620 nm, the false color scale is indicated below the respective FLIM images in (a, c).

We next attempted FLIM to study **TO** and **ThT** in live cells. Both dyes (at 2–5 μM) were incubated with cells for 1–24 h prior to imaging; bright fluorescence staining with characteristic nuclear and nucleolar staining was achieved in all cases. Our FLIM experiments for both dyes (Figures 3, S11, and S14) showed that the cellular lifetime was in between benchmark *in vitro* lifetimes characteristic of duplex and G4 DNA for both dyes.

However, for **TO** we observed a systematic increase in fluorescence intensity but a decrease in fluorescence lifetime in U2OS cell nuclei over time (Figure S12). These changes are consistent with the concentration-dependent aggregation of **TO**. As a result of excessive dye internalization in cells over time, intracellular fluorescence of **TO** increases; however, increasing concentration of the dye enables aggregate formation, which are characterized by a short lifetime. We were not able to find experimental conditions to record a stable lifetime of **TO** in live cells, even at lower incubation concentrations and short incubation times. Due to the poor reproducibility of FLIM data in live cells, we discarded **TO** as a fluorescence lifetime probe for G4 DNA visualization in live cells.

**ThT**, on the other hand, showed high fluorescence counts (Figure S13) and a stable FLIM lifetime in live cells under various staining conditions, giving us confidence that no aggregates were present. **ThT** shows nuclear as well as cytosolic localization in live and fixed U2OS cells, with preferential localization in the nucleoli, Figure 3. The average lifetime recorded from the whole nuclei in live and fixed cells, τ_w_ of 1.87 ns (5–95% Cl 1.71–2.10 ns) is consistent with **ThT** binding to a mixture of duplex/G4 DNA, as the lifetimes fall between those characteristics of duplex DNA and G4 DNA, measured *in vitro* (Figure 1b). FLIM analysis showed a consistently higher lifetime from nucleoli of live U2OS cells compared to the whole nucleus, Figures 3a and S12b. There is evidence that G4s are enriched in nucleoli, as it is the site of ribosomal RNA (rRNA) transcription which is highly G-rich.^32^ Also, our recent study using a probe suitable for phosphorescence lifetime imaging microscopy (PLIM) has found a considerably higher lifetime in nucleoli, compared to the whole nucleus, indicating a higher nucleolar concentration of G4s.^15^ The mitochondria of live cells are also stained by the probe; however, they display a lower lifetime, *ca*. 1.1 ns, which is inconsistent with exclusive DNA binding of the probe, Figure 3c.

Our data is consistent with **ThT** binding to a fraction of G4 in nuclei of cells, however, other factors of the cellular environment can contribute to the observed lifetime, such as higher-level chromatin organization.^33^ We sought to further confirm that a fraction of **ThT** is bound to G4 in nuclei by attempting to displace it with a known G4 binder, Ni-Salphen^20^ (Figure 3c–e).

*In vitro*, the addition of Ni-Salphen causes a **ThT** displacement from G4 manifested as a large decrease in lifetime, eventually reaching the values seen in duplex DNA (Figure S9). Previously, we used Ni-Salphen displacement to demonstrate that our lifetime-based G4 probes were indeed localized to G4s in cells.^13,15,16^ As expected, while the nuclear staining of **ThT** remained following incubation with this G4 binder (although the balance between nuclear, nucleolar and mitochondrial intensity is changed), the lifetime observed in all organelles was reduced, when incubated alongside 1 μM Ni-Salphen for 1 h, specifically in nuclei the lifetime was reduced to 1.43 ns (Figure 3e). This data is consistent with **ThT** being displaced from G4s into duplex DNA in nuclei of cells.

### ThT as a G4 RNA Probe

Given the nucleolar localization of **ThT**, which is rich in RNA, we wanted to ascertain whether the probe is bound to G4 DNA or RNA. **ThT** displays long lifetimes upon binding to G4 RNA *in vitro* (Figure S10 and Table S6), in line with lifetimes seen upon G4 DNA binding, Figure 1. Consequently, fixed cells were incubated with **ThT** and treated with DNase or RNase A to selectively digest these nucleic acids (Figures 3a and S14a). Initially, we confirmed that the lifetimes and staining patterns of **ThT** seen in untreated fixed cells are comparable to those of live cells, Figure 3. This data indicates that a similar proportion of G4 and duplex DNA is stained by **ThT** under both conditions. Thus, the nuclease treatment results (that are only possible in fixed cells) are relevant for G4 content in live cells.

Incubation with both RNase and DNase caused a statistically significant change in the lifetime of **ThT** from nuclei. A change in the staining pattern, resulting in no nucleolar staining, was seen with RNase, as well as a reduction in lifetime down to 1.76 ns (5–95% Cl 1.67–1.85 ns). The reduction in lifetime is consistent with the reduced number of G4s being stained after the RNase treatment. The change in staining pattern is consistent with the facts that (i) a significant proportion of RNA is localized in nucleoli and (ii) **ThT** preferentially stained RNA in nucleoli before RNase treatment. We note that even after the RNase treatment the **ThT** lifetime seen in the nuclei of fixed cells is *ca*. 1.76 ns, which is in-between the typical lifetimes of duplex and quadruplex DNA seen *in vitro* (Figure 1). It also significantly exceeds the lifetime seen upon Ni-Salphen treatment of live cells, 1.40 ns (5–95% Cl 1.29–1.57 ns), which was expected to result in the complete removal of **ThT** from cellular G4s. Thus, these data are consistent with **ThT** staining both DNA and RNA G4s in cells, with most of the RNA G4s localized in the nucleoli of nuclease-untreated cells.

The opposite trend in lifetime was seen upon the DNase treatment of fixed cells: bright nucleolar staining pattern remained, and an increased lifetime was detected: 1.98 ns (5–95% Cl 1.92–2.05 ns). This change is consistent with increased numbers of G4s being stained upon removal of DNA from cells. Since our RNase treatment data indicated that a mixture of DNA duplex and G4s were stained by **ThT** in the nucleus (not the nucleoli) of cells, we hypothesize that upon DNA removal some of the **ThT** is forced to relocalize from duplex DNA to G4 RNA thus increasing the lifetime. We note that the intensity contrast between whole nuclei and nucleoli reduced upon DNase treatment, thus indicating that while nucleolar G4 RNA remained the most intensely stained organelle, overall nuclear RNA G4 staining increased.

Overall, we interpret our data as **ThT** preferentially binding to G4 RNA in nucleoli (which are removed upon RNase treatment in favor of duplex DNA). Simultaneously, **ThT** binds to a mixture of duplex and G4 DNA in the whole nucleus. The duplex staining is removed upon DNase treatment in favor of RNA G4s. This data can be compared to a recent report by us^16^ of a **TO**-based dye that selectively and exclusively stained G4 RNA in cells, and others, which showed dual DNA/RNA G4 staining.^18^

## Conclusions

In summary, our data indicate that both commercially available dyes, **TO** and **ThT**, display distinct fluorescence lifetimes upon binding to different DNA topologies *in vitro*. Both dyes show intense fluorescence staining of live cells. However, only **ThT** worked successfully as an FLIM-based probe for G4 DNA/RNA detection in live cells. We believe that further advances in sensitivity and selectivity can be achieved by investigating a diverse range of **ThT** derivatives and studying their cellular behavior *via* FLIM. Most importantly, the combined use of commercially available **ThT** and FLIM should allow quantitative detection of G4s to a wider biophysical community, allowing elucidation of their essential biological roles more widely.

## Supporting Information Available

Photophysical characterization including absorbance and fluorescence spectra and time-resolved fluorescence decay traces of **TO** and **ThT**; method of fitting average weighted fluorescence lifetime, components, and goodness of fit when dyes are bound to nucleic acids or upon changes of viscosity; statistical analysis of data. Aggregation of **TO** in cells; *in vitro* displacement assays; fluorescence intensity and lifetime imaging of U2OS cells at conditions of staining and fixation; DNase/RNase treatment and displacement of G4 bound probe; and fluorescence time-resolved decays of **ThT** in cells (PDF)
