# Supplementary material for: Imaging G-Quadruplex Nucleic Acids in Live Cells Using Thioflavin T and Fluorescence Lifetime Imaging Microscopy
Source: Anal Chem. 2024 Dec 11;96(51):20223–9. doi: 10.1021/acs.analchem.4c04207 (PMC11672229; doi:10.1021/acs.analchem.4c04207)
Supplement: Supplementary file 1 — ac4c04207_si_001.pdf [file ac4c04207_si_001.pdf]

Supporting Information

Imaging G-Quadruplex nucleic acids in live cells using Thioflavin T and fluorescence lifetime imaging microscopy.

Tigerlily Bradford,[a] Peter A. Summers,[a] Aatikah Majid,[a] Petr S. Sherin,[a] Jeff Yui Long Lam,[a] Savyasanchi Aggarwal,[a] Jean-Baptiste Vannier,[b,c] Ramon Vilar[a]*, Marina K. Kuimova[a]*

[a]Department of Chemistry, Molecular Sciences Research Hub, 82 Wood Lane, White City Campus, Imperial College London, W12 0BZ, UK.

[b]Telomere Replication and Stability group, Medical Research Council – London Institute of Medical Sciences, London, W12 0NN, UK.

[c]Institute of Clinical Sciences, Faculty of Medicine, Imperial College London, London, W12 0NN, UK.

Corresponding authors: m.kuimova@imperial.ac.uk; r.vilar@imperial.ac.uk

Table of Content

Methods	S2
Additional spectroscopic data	S3-S10
Additional imaging data	S11-S13
Notes and references	S13

Methods

Table S1: List of all the DNA/RNA oligonucleotides used, their corresponding sequence and their extinction coefficients in $M^{-1}cm^{-1}$.

DNA	Sequence	Extinction Coefficient (M^{-1} cm^{-1})
c-Myc	TGAGGGTGGGTAGGGTGGGTAA	228700
c-kit87up	AGGGAGGGCGCTGGGAGGAGGG	226700
Myc-2345	TGAGGGTGGGGAGGGTGGGGAA	229900
HTG4	AGGGTTAGGGTTAGGGTTAGGG	228500
ds26	CAATCGGATCGAATTCGATCCGATTG	253200
ds17 s1, s2	CCAGTTCGTAGTAACCC, GGGTTACTACGAACTGG	160900, 167400
ss17s1	CCAGTTCGTAGTAACCC	160900
ATss17s1	CTATTGCATACTAATTC	161000
TRF2	CGGGAGGGCGGGGAGGGC	166 615

DNA oligonucleotides were purchased from Eurogentec (RP Cartridge purification) and used as received. The oligonucleotides were dissolved in 10 mM lithium cacodylate buffer at pH 7.3. KCl was added to a final concentration of 100 mM, and the resulting oligonucleotide solution was annealed at 95 °C for 10 min. Calf thymus DNA (ctDNA, Sigma) was dissolved in the same cacodylate buffer, and KCl added to a final concentration of 100 mM. All oligonucleotide concentrations were determined in salt free buffer (before any annealing) using the molar extinction coefficients listed in Table S1 and 13200 M^{-1} cm^{-1} (base pair for ctDNA). Concentrations of G4s are per strand while concentrations of dsDNA are per base pair. Single strands of ds17 DNA were combined at a molar ratio of 1:1 to make double-stranded ds17.

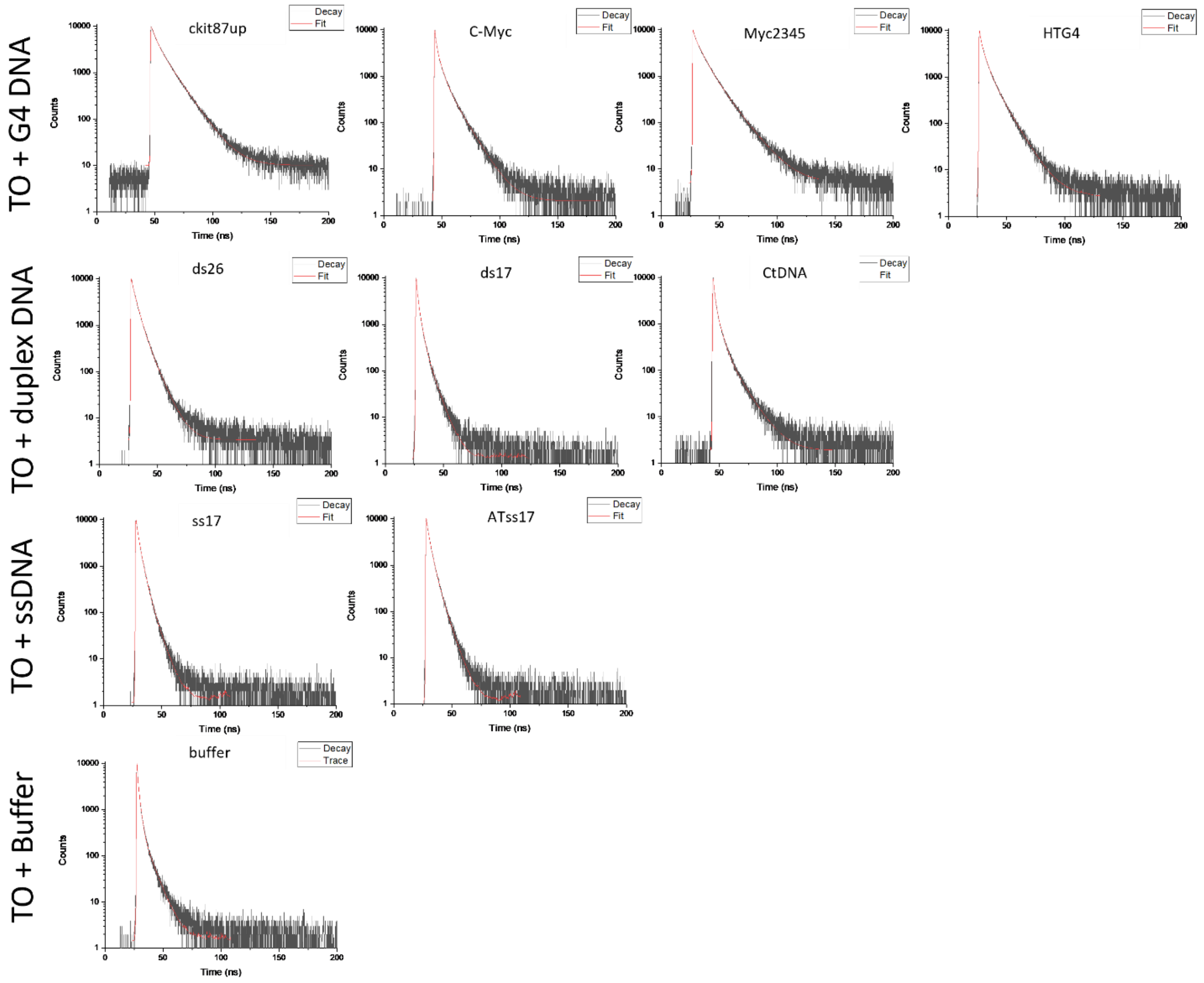

Figure S1: Representative decay traces for 2 µM **TO** (λex = 467 nm, λem = 530±8 nm); upon addition of 10 µM of G4 DNA (top row) and 40 µM of duplex (middle row) and single stranded DNA. The data for **TO** in the buffered solution is also shown (bottom row). The resultant fitting parameters are shown in Table S2.

Table S2: Representative decay bi and tri-exponential fitting parameters for 2 µM **TO** (λex = 467 nm, λem = 530±8 nm); upon addition of 40 µM of duplex and single stranded DNA and 10 µM of G4 DNA. The corresponding decay traces are shown in Figure S1.

		**a1**	**t1**	**a2**	**t2**	**a3**	**t3**	**Chi Sq.**	**Tw**
TO	**ckit87up**	0.42	0.48	0.38	3.12	0.2	5.81	1.22	4.07
TO	**C-Myc**	0.44	1.26	0.58	4.7			1.41	3.98
TO	**myc2345**	0.37	0.25	0.53	3.11	0.1	6.73	1.48	4.00
TO	**HTG4**	0.43	0.48	0.39	2.71	0.17	5.88	1.22	3.93
TO	**CtDNA**	0.7	0.45	0.26	1.24	0.04	5.42	1.70	1.95
TO	**ds26**	0.39	0.50	0.51	1.90	0.10	4.01	1.17	2.27
TO	**ds17**	0.79	0.24	0.19	1.37	0.02	4.49	1.17	1.54
TO	**ss17**	0.06	0.02	0.80	0.09	0.13	0.11	1.22	0.09
TO	**atss17**	0.86	0.20	0.13	1.21	0.01	3.62	1.22	0.87
TO	**buffer**	0.96	0.03	0.03	0.25	0.01	1.29	1.01	0.11

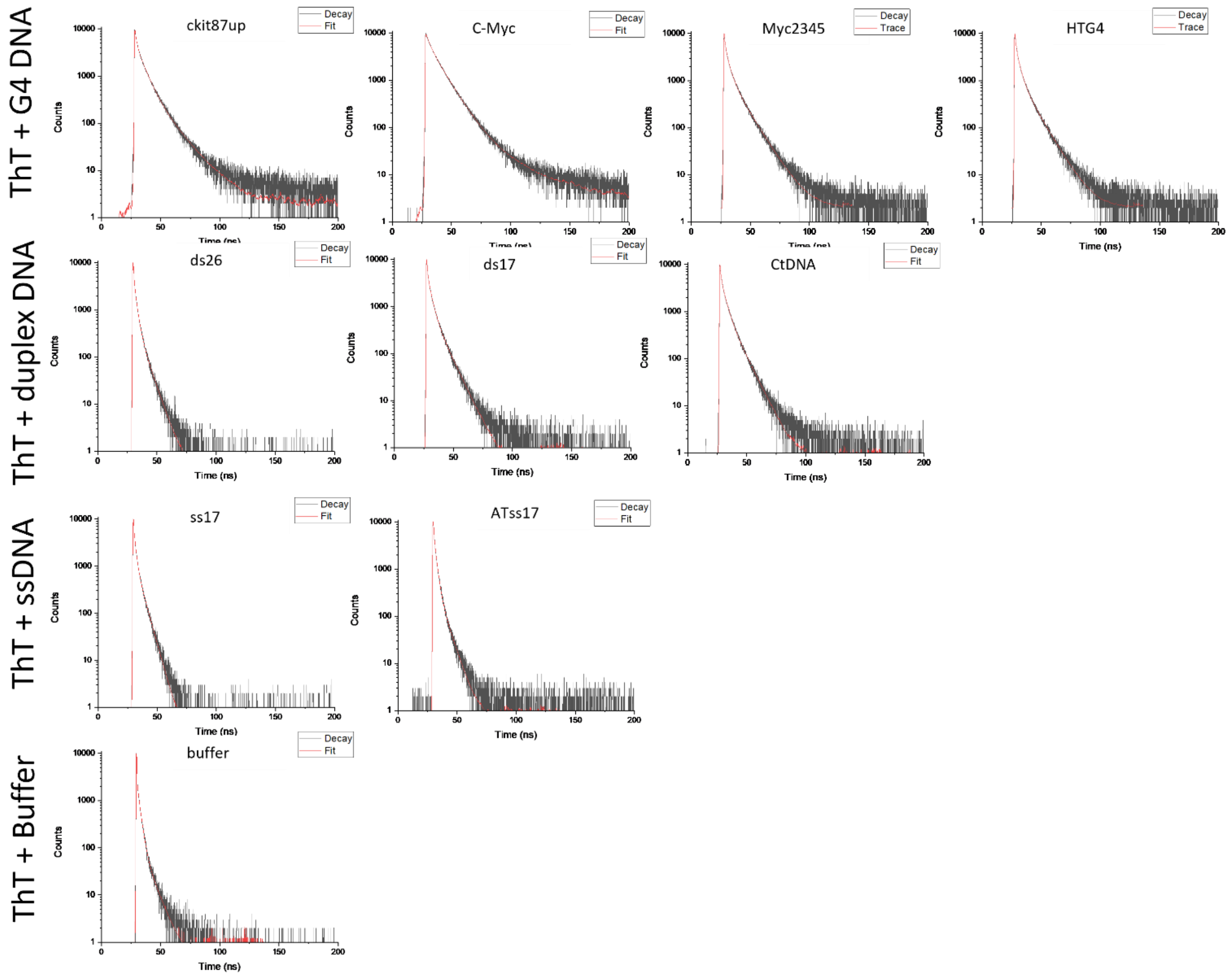

Figure S2: Representative decay traces for 2 µM **ThT** (λex = 405 nm, λem = 490±8 nm) upon addition of 10 µM of G4 DNA (top row) and 40 µM of duplex (middle row) and single stranded DNA. The data for **ThT** in the buffer solution is also shown (bottom row). The resultant fitting parameters are shown in Table S3. We cannot exclude a possible effect of small undetected quantities of aggregated dyes in aqueous solutions as a contributing factor towards high error bars seen in intensity-based data, Figures 1a and 1d. Additionally, we note that fluorescence enhancement seen for ThT with G4s (x 200-400) compared to duplex DNA (x 50) is much larger compared to the lifetime enhancement (ca 2.4 ns for G4 compared to 1.2 ns for duplex DNA). We suggest that this is due to small undetected quantities of aggregated ThT in aqueous solutions, as previously reported,[3] that may affect the absorption spectra of the dye, thus causing a mismatch.

Table S3: Representative decay bi and tri-exponential fitting parameters for 2 µM **ThT** (λex = 405 nm, λem = 490±8 nm); upon addition of 40 µM of duplex and single stranded DNA and 10 µM of G4 DNA. The corresponding decay traces are shown in Figure S2.

		**a1**	**t1**	**a2**	**t2**	**a3**	**t3**	**Chi Sq.**	**Tw**
**ThT**	**ckit87up**	0.22	0.92	0.64	2.40	0.14	3.89	1.19	2.61
**ThT**	**C-Myc**	0.48	0.45	0.33	1.48	0.19	3.74	1.04	2.44
**ThT**	**myc2345**	0.53	0.50	0.22	0.65	0.25	3.34	1.16	2.43
**ThT**	**HTG4**	0.43	0.35	0.38	1.79	0.19	3.21	1.05	2.24
**ThT**	**CtDNA**	0.76	0.26	0.22	1.34	0.02	3.52	1.05	1.23
**ThT**	**ds26**	0.59	0.18	0.28	0.87	0.13	2.04	1.41	1.26
**ThT**	**ds17**	0.29	0.19	0.55	0.85	0.16	2.14	1.06	1.32
**ThT**	**ss17**	0.23	0.11	0.43	0.68	0.01	2.4	1.60	0.79
**ThT**	**atss17**	0.78	0.23	0.2	0.63	0.02	1.4	1.07	0.47
**ThT**	**buffer**	0.97	0.01	0.02	0.02	0.01	0.42	1.09	0.13

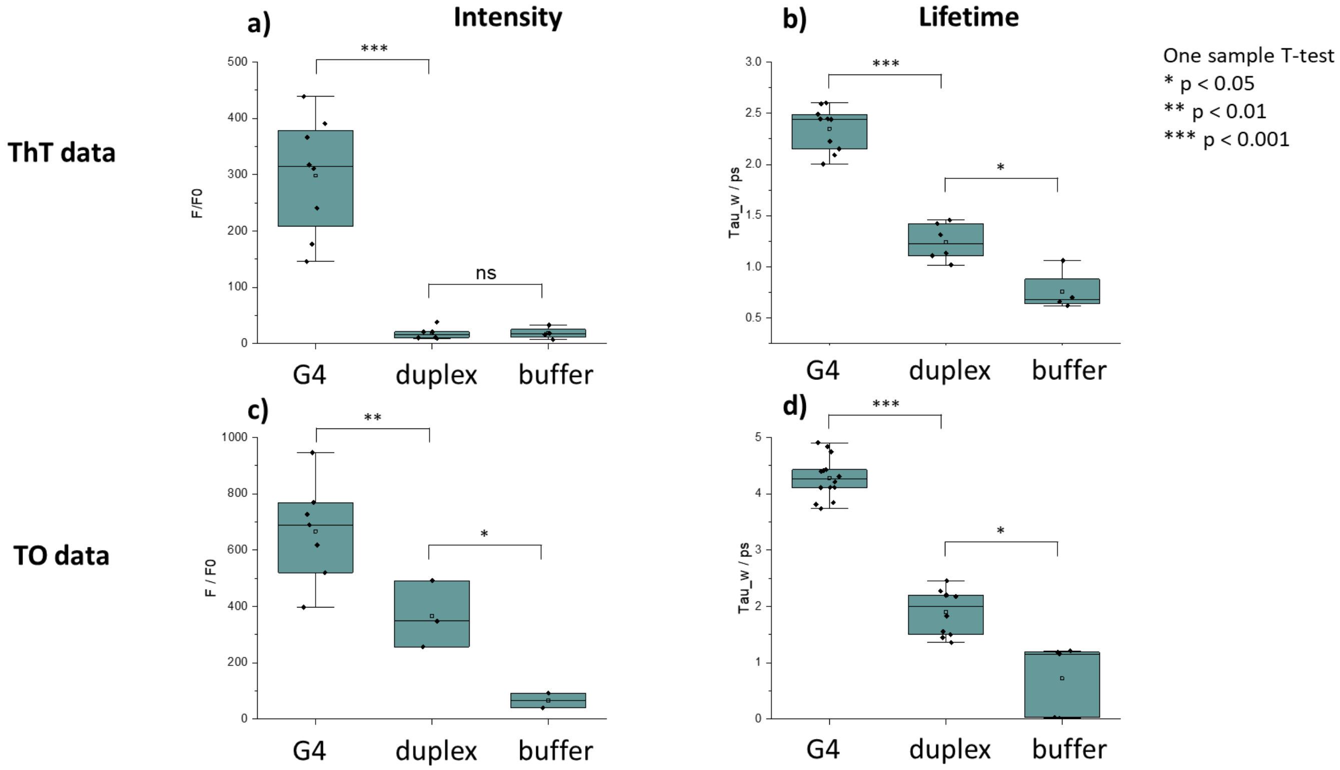

Figure S3: Statistical analysis of the spectroscopic data for **ThT** and **TO** presented in the main text, Figure 1, *via* one sample T-test. **ThT** data (top, a, b) and **TO** data (bottom (c, d); intensity data (left, a, c) and lifetime data (right, b, d). Each independent repeat is represented by a separate data point, * p < 0.05; ** p < 0.01; *** p < 0.001. The lifetimes recorded for **ThT** and **TO** are highly statistically significant (p < 0.001) for G4 vs duplex DNA ((b, d). While the fluorescence enhancement in the presence of G4 vs duplex is highly statistically significant (p < 0.001) for **ThT** (a); for the fluorescence enhancement in **TO**, in the presence of G4 vs duplex DNA p < 0.01 was obtained.

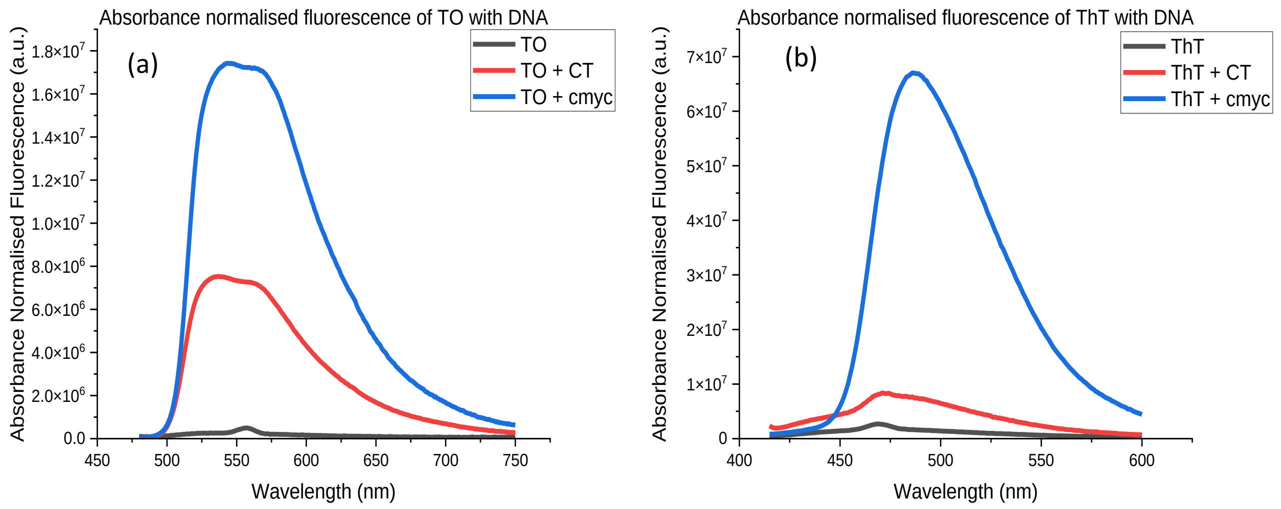

Figure S4: Fluorescence spectra of **TO** (a) and **ThT** (b) free in the buffered aqueous solution (black) and upon interaction with 10 mM G4 (c-Myc, blue) and 40 mM duplex DNA (CtDNA, red). For 2 µM **ThT**, λex = 404 nm, for 2 µM **TO**, λex = 467 nm. All experiments in 10 mM lithium cacodylate buffer (pH7.3) with 100 mM KCl. We cannot exclude a possible effect of small undetected quantities of aggregated dyes in aqueous solutions as a contributing factor towards high error bars seen in intensity-based data, Figures 1a and 1d. Additionally, we note that fluorescence enhancement seen for ThT with G4s (x 200-400) compared to duplex DNA (x 50) is much larger compared to the lifetime enhancement (ca 2.4 ns for G4 compared to 1.2 ns for duplex DNA). We suggest that this is due to small undetected quantities of aggregated ThT in aqueous solutions, as previously reported,[3] that may affect the absorption spectra of the dye, thus causing a mismatch.

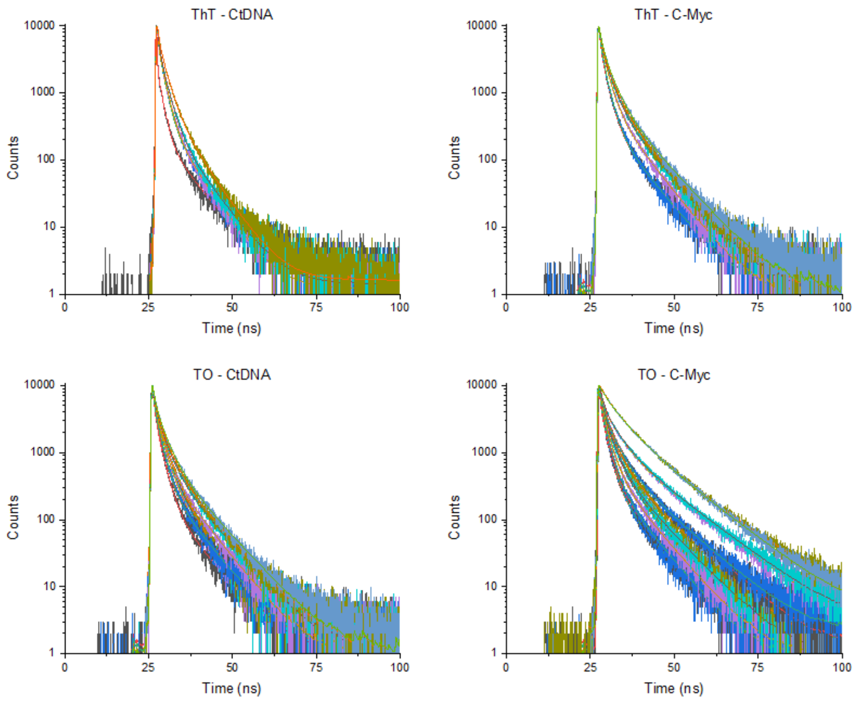

Figure S5: Sample time resolved decays obtained for 1 µM **ThT** (top, λex = 405 nm, λem = 490±8 nm) and 1 µM **TO** (bottom, λex = 467 nm, λem = 530±8 nm); upon addition of duplex (CtDNA, left) and quadruplex (G4, right) DNA solutions from stock between 0-50 µM DNA. The averaged lifetime data from three independent experiments is shown in Figure S5 below. All *in vitro* experiments in 10 mM lithium cacodylate buffer (pH7.3) with 100 mM KCl.

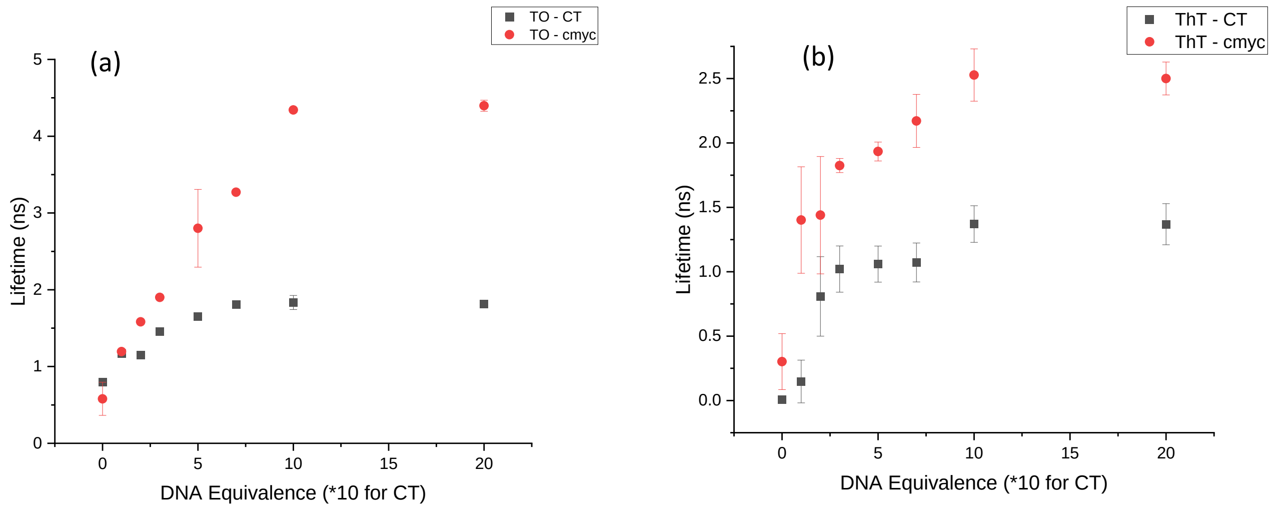

Figure S6: The effect of addition of c-Myc (G4, red) and CT (duplex, black) DNA on the weighted average lifetime of (a) **TO** and (b) **ThT.** The conditions were 1 µM **TO**, λ_{ex} = 467 nm, λ_{em} = 530±8 nm; and 1 µM **ThT**, λ_{ex} = 404 nm, λ_{em} = 490±8 nm; 50 µM of c-Myc DNA and 500 µM of CtDNA were used as stocks and diluted to achieve the tested concentrations. All experiments completed in 10 mM lithium cacodylate buffer (pH7.3) with 100 mM KCl. An average of three independent experiments is shown.

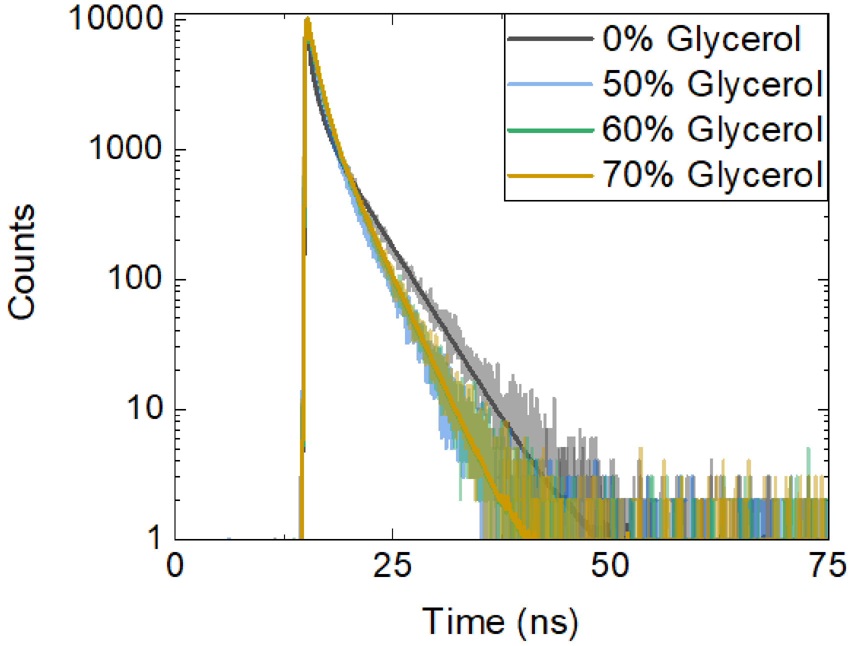

Figure S7: Sample time-resolved decay traces obtained for 2 µM **ThT** (λex = 405 nm, λem = 490±8 nm) with 2 µM of c-Myc DNA in solutions of varying viscosity, made from 0% - 70% glycerol in water. The traces recorded in 50-70% glycerol overlap completely, while 0% glycerol shows small changes. However, the intensity-weighted average lifetime from these traces is the same, Table S4. This data allows us to rule out the effect of elevated crowding/viscosity as the determining factor of cellular lifetime of **ThT**.

Table S4: Representative decay tri-exponential lifetime components for 2 µM **ThT** (λex = 405 nm, λem = 490±8 nm); with 2 µM of c-Myc DNA in solutions of varying viscosity, made from 0% - 70% glycerol in water The corresponding decay traces are shown in Figure S6.

**% Glycerol**	**Approx viscosity (cP)**	**a1**	**t1 (ns)**	**a2**	**t2 (ns)**	**a3**	**t3 (ns)**	**Chi Sq.**	**t_w (ns)**
**0**	0.89	0.11	0.11	0.36	0.93	0.53	3.11	0.99	2.72
**50**	10.37	0.11	0.06	0.40	1.03	0.48	3.01	1.02	2.56
**60**	20.6	0.11	0.05	0.44	1.04	0.46	3.02	1.03	2.53
**70**	46.99	0.11	0.03	0.44	1.01	0.45	3.02	1.05	2.52

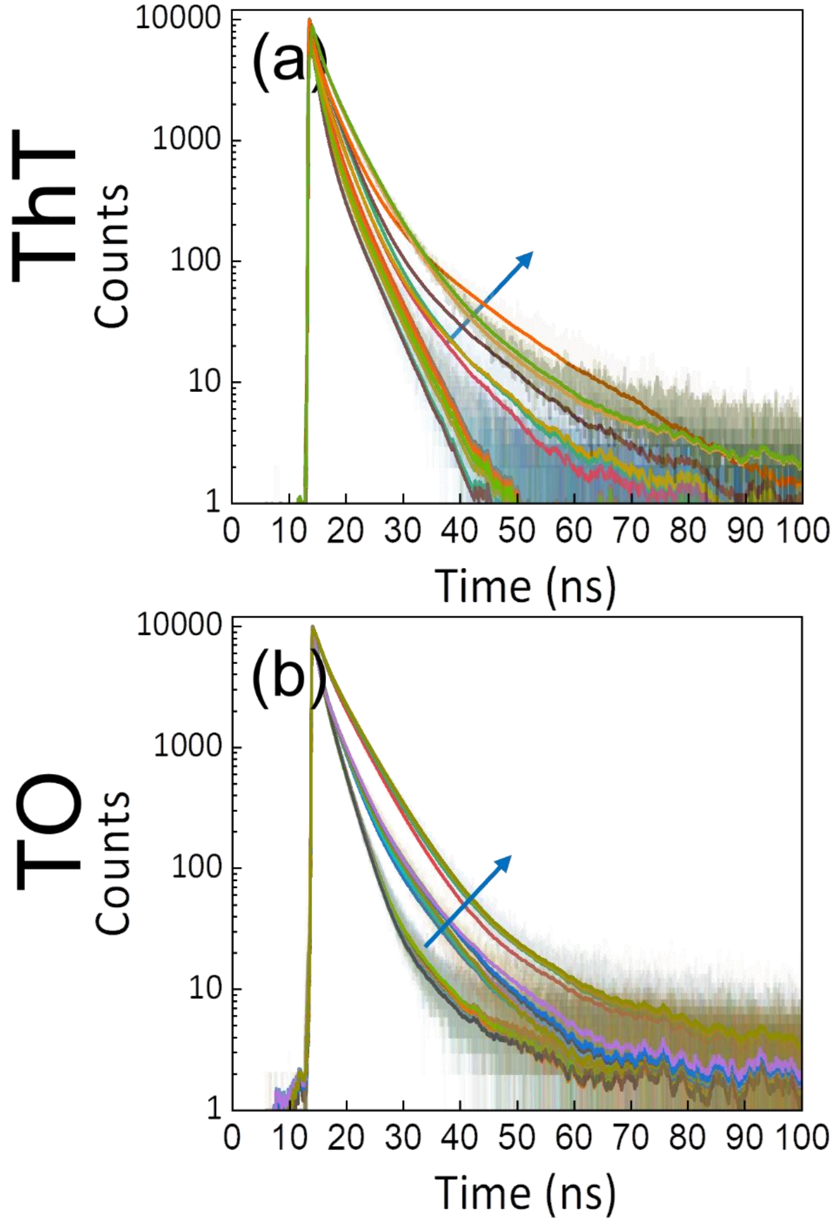

Figure S8: The effect of G4:duplex DNA ratio on the time-resolved fluorescence decays of **ThT** and **TO** (a) 2 µM ***ThT,*** λ_{ex} = 404 nm, λ_{em} = 490±32 nm and in (b) 2 µM **TO**, λ_{ex} = 467 nm, λ_{em} = 530±32 nm, with arrows representing increasing G4 concentration, from 10^{-4}:1 to 10:1 C-Myc DNA:CtDNA. The [dye]:DNA ratio was maintained at 1:20 per G4 (C-Myc) or per bp (CtDNA). In the case of **ThT**, its binding and consequent fluorescence enhancement in the presence of G4 is high and, therefore, even small concentrations of G4 are sufficient to produce a shift in lifetime, compared to the pure duplex DNA lifetime.

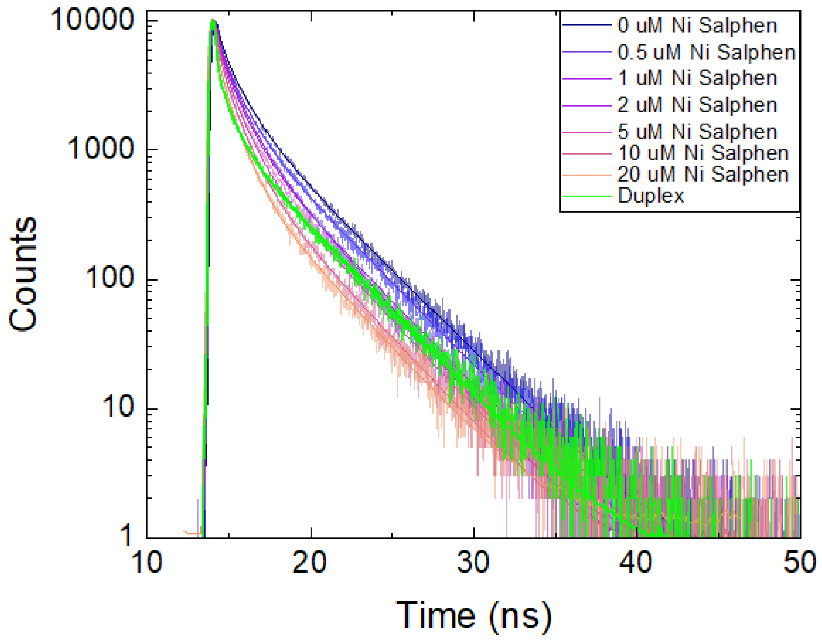

.

Figure S9: Sample time-resolved decays obtained for 2 µM **ThT** (λex = 404 nm, λem = 490±8 nm) in the presence of 2 µM of c-Myc DNA and 200 µM of CtDNA upon the addition of increasing concentrations of Ni-Salphen. A gradual decrease in **ThT** lifetime is seen upon increasing Ni-Salphen. This is consistent with the displacement of **ThT** from G4 to duplex DNA.

A trace of 2 µM **ThT** in the presence of duplex DNA (40 µM CtDNA) is also shown for comparison.

Table S5: Representative decay bi and tri-exponential lifetime components for 2 µM **ThT** (λex = 405 nm, λem = 490±8 nm); in in solutions of varying Ni-Salphen concentration. The corresponding decay traces are shown in Figure S8.

**Ni Salphen/ µM**	**a1**	**t1 (ns)**	**a2**	**t2 (ns)**	**a3**	**t3 (ns)**	**Chi Sq.**	**t_w (ns)**
0.00	0.57	0.23	0.34	1.14	0.10	4.44	0.91	2.50
0.10	0.58	0.25	0.33	1.13	0.08	4.38	0.98	2.34
0.50	0.60	0.26	0.33	1.10	0.08	4.14	1.00	2.11
1.00	0.60	0.26	0.33	1.10	0.06	4.04	0.99	1.91
2.00	0.65	0.24	0.30	1.11	0.05	4.04	1.04	1.82
5.00	0.68	0.18	0.27	1.01	0.05	3.88	1.18	1.74
10.00	0.81	0.17	0.17	1.08	0.02	3.13	1.63	1.13
20.00	0.82	0.19	0.16	1.16	0.02	3.07	1.55	1.08
duplex	0.33	0.18	0.43	0.75	0.33	1.90	1.34	1.44

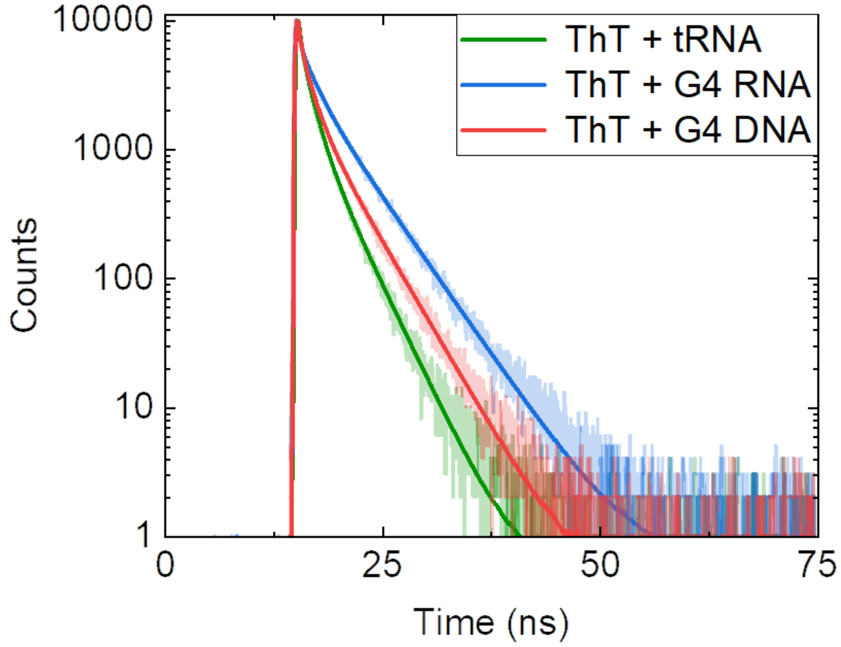

Figure S10: Sample time-resolved decays obtained for 2 µM **ThT** (λex = 405 nm, λem = 490±8 nm) upon addition of 2 µM G4 DNA Myc2345 (red) and 1:10 by mass G4 RNA TRF2 (blue) and tRNA (green).

Table S6: Representative decay bi and tri-exponential lifetime components for 2 µM ThT (λex = 405 nm, λem = 490±8 nm); in various DNA and RNA sequences at various conditions. The corresponding decay traces are shown in Figure S10.

**Sequence**	**a1**	**t1 (ns)**	**a2**	**t2 (ns)**	**a3**	**t3 (ns)**	**Chi Sq.**	**t_w (ns)**
**G4 RNA (TRF2)**	0.61	0.23	0.19	1.74	0.20	4.51	1.34	3.40
**G4 DNA (Myc2345)**	0.56	0.12	0.30	1.51	0.14	3.63	1.15	2.48
**tRNA**	0.51	0.27	0.36	1.16	0.13	3.05	1.10	1.82

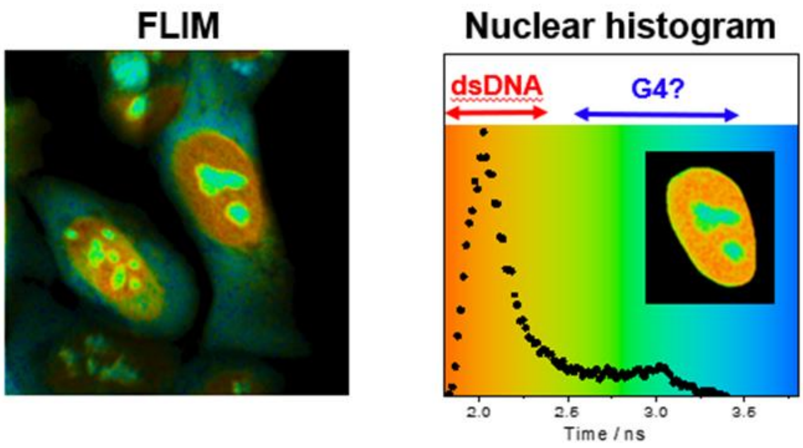

Figure S11: A FLIM image of live U2OS cells incubated with **TO** at 2μM concertation for 24 hours and the corresponding lifetime frequency histogram of the individual segmented nucleus (image is shown as an insert); λ_{ex} = 477 nm, λ_{em} = 550–700 nm, the false colour scale is from 1.9 ns (orange) to 3.8 ns (blue). The lifetimes characteristic of **TO** binding to both ds DNA and G4 DNA are observed in cellular FLIM images (see Fig 1 main text for the values).

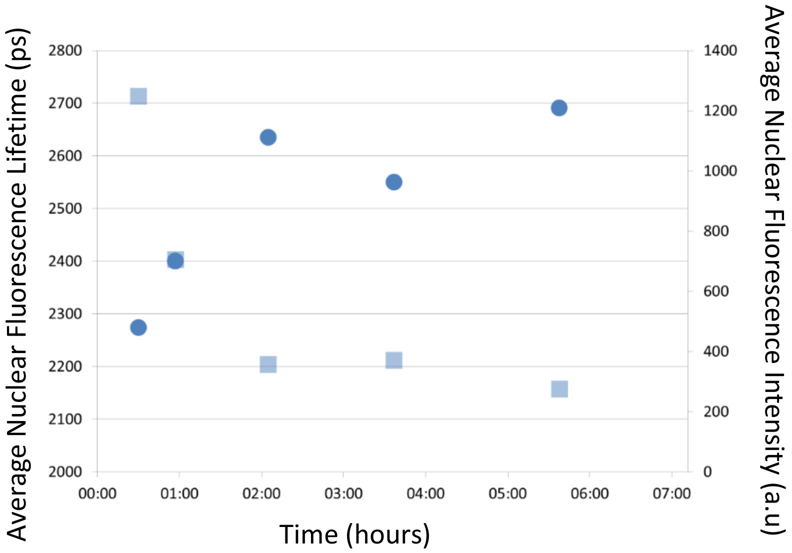

Figure S12: The results of FLIM image analysis for whole unsegmented U2OS cells incubated with **TO** at 2 μM concentration for 0.5-6h, λ_{ex} = 477 nm, λ_{em} = 550–700 nm. The average lifetimes of **TO** within each image (square symbols, left axis) show a decrease as a function of incubation time, while the intensity within each image (circle symbols, right axis) shows a significant increase. Thus, two processes occur in cells upon incubation with **TO**: higher uptake of the probe (leading to higher fluorescence intensity), and aggregation, caused by high concentration (leading to shorter lifetime). This data is consistent with aggregation of **TO** in cells as a function of incubation time.

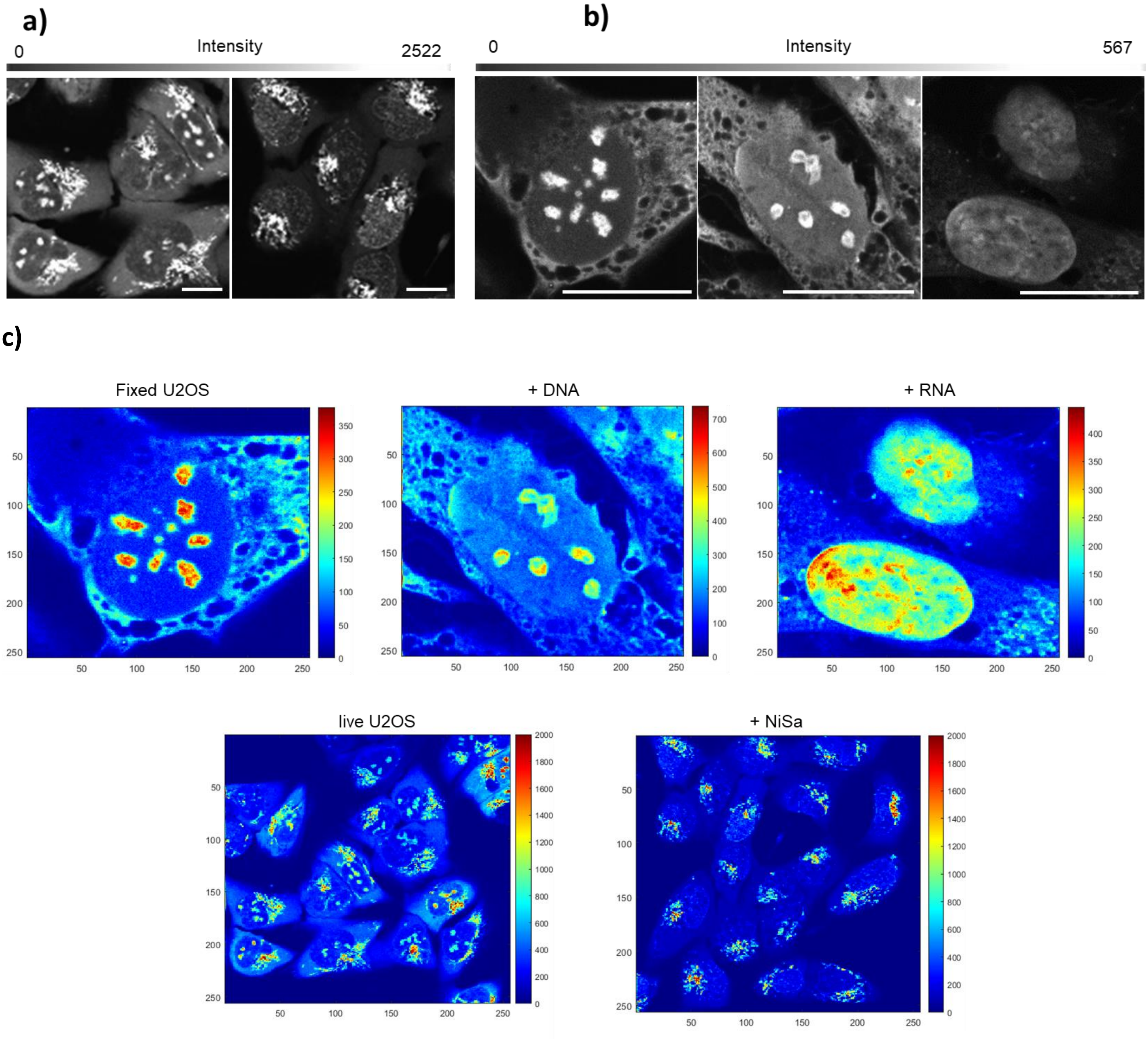

Figure S13: Fluorescence intensity per pixel distribution from U2OS cells incubated for 1 hour with 5 µM **ThT**, λ_{ex} = 850 nm, λ_{em} = 440–620 nm, scale bar 20 µm; (a-b) grey scale and c) false colour scale produced in Matlab: (a) live cells before (left) and after (right) the treatment with Ni-Salphen and (b) fixed cells untreated/treated with DNase/treated with RNase (left to right). c) individual images are labelled with the staining conditions. The fixation was responsible for the significantly reduced intensity seen per pixel in b).

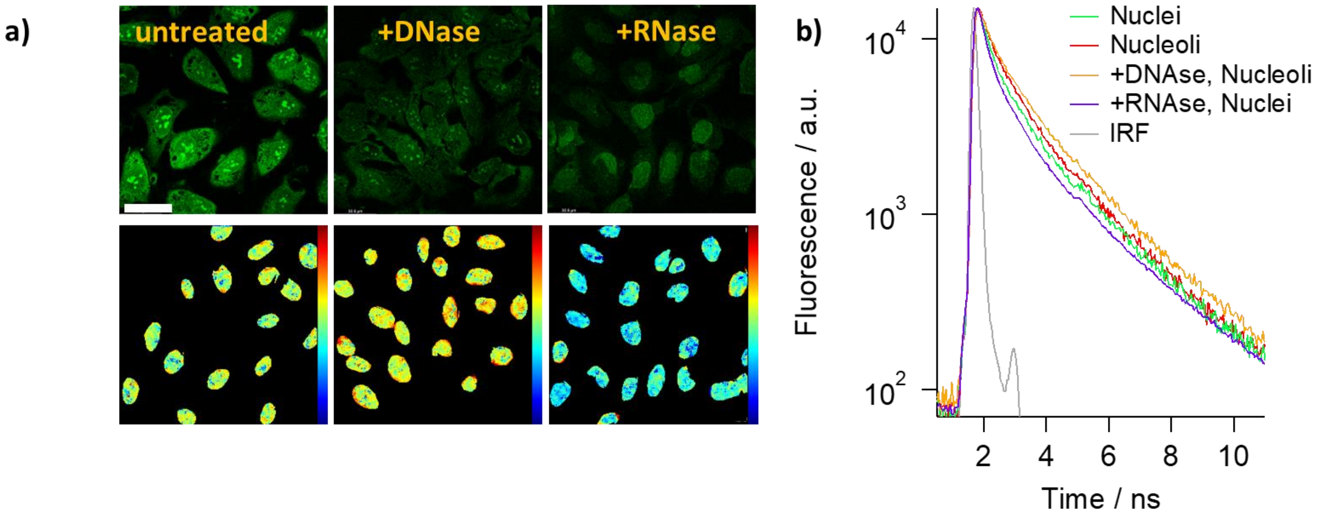

Figure S14: Confocal (top) and FLIM (bottom) data from U2OS cells incubated for 1 hour with 5 µM **ThT**. (a) images of fixed cells that were untreated or incubated with DNase and RNase A, the false colour scale is from 1.24 ns (blue) to 2.42 ns (red) for all FLIM images, scale bar 30.9 µm; (b) Time-resolved traces recorded at various conditions shown in a) λ_{ex} = 850 nm, λ_{em} = 440–620 nm. DNase causes an increase in lifetime seen in the nuclei and nucleoli, while RNase causes a lifetime decrease.

Notes and references

1. O. Suss, L. Motiei, D. Margulies, *Molecules*, 2021, **26**, 2828.
2. S. Das and P. Purkayastha, *ACS Omega*, 2017, **8**, 5036-5043
3. X. Christine, L. T.Yuwen, C. Dennis and G. Zhefeng, *R. Soc. Open Sci,* **4,** 160696-160696
